# Clinical Outcomes and Virulence Factors of Shiga Toxin-Producing *Escherichia coli* (STEC) from Southern Alberta, Canada, from 2020 to 2022

**DOI:** 10.3390/pathogens13100822

**Published:** 2024-09-24

**Authors:** Heather Glassman, Vivien Suttorp, Theron White, Kim Ziebell, Ashley Kearney, Kyrylo Bessonov, Vincent Li, Linda Chui

**Affiliations:** 1 Department of Laboratory Medicine and Pathology, University of Alberta, Edmonton, AB T6G 2R3, Canada; 2 Medical Officer of Health, Alberta Health Services, Lethbridge, AB T1J 4E1, Canada; 3 Environmental Public Health, South Zone, Alberta Health Services, Taber, AB T1G 1N9, Canada; 4 National Microbiology Laboratory at Guelph, Public Health Agency of Canada, Guelph, ON N1G 3W4, Canada; 5 National Microbiology Laboratory, Public Health Agency of Canada, Winnipeg, MB R3C 4W1, Canada; ashley.kearney@phac-aspc.gc.ca; 6 Alberta Precision Laboratories-Public Health Laboratory, Edmonton, AB T6G 2J2, Canada

**Keywords:** Shiga toxin-producing *Escherichia coli*, virulence factors, whole genome sequencing

## Abstract

Shiga toxin-producing *Escherichia coli* (STEC) can cause severe clinical disease in humans, particularly in young children. Recent advances have led to greater availability of sequencing technologies. We sought to use whole genome sequencing data to identify the presence or absence of known virulence factors in all clinical isolates submitted to our laboratory from Southern Alberta dated 2020–2022 and correlate these virulence factors with clinical outcomes obtained through chart review. Overall, the majority of HUS and hospitalizations were seen in patients with O157:H7 serotypes, and HUS cases were primarily in young children. The frequency of virulence factors differed between O157:H7 and non-O157 serotypes. Within the O157:H7 cases, certain virulence factors, including *espP*, *espX1*, and *katP*, were more frequent in HUS cases. The number of samples was too low to determine statistical significance.

## 1. Introduction

Shiga toxin-producing *Escherichia coli* (STEC) are a group of foodborne enteric pathogens that can cause hemorrhagic colitis and hemolytic uremic syndrome (HUS). HUS, composed of a triad of microangiopathic hemolytic anemia, thrombocytopenia, and renal failure, is most common in children less than 5 years of age. It can lead to severe clinical outcomes, including renal failure, dialysis dependence, and death [1].

Whole genome sequencing (WGS), previously prohibitively expensive and labor-intensive, has now expanded to the clinical laboratory. Historically, clinical STEC isolates were primarily tested for the presence of Shiga toxins and their subtypes, which have been correlated with virulence [2]. However, Shiga toxins alone do not fully account for STEC virulence potential. Other virulence factors include the locus of enterocyte effacement (LEE) pathogenicity island, which has also been associated with hemorrhagic colitis and HUS [3]. A host of other virulence factors have also been identified in the research literature but are not identified routinely in clinical isolates.

In Alberta, all STEC isolates from the entire province are submitted to Alberta Precision Laboratory—Public Health Laboratory: ProvLab for molecular serotyping, cluster analysis, and surveillance by WGS. This testing and analytics provide a wealth of new epidemiological information on STEC. For example, databases are available to analyze these data for the presence or absence of known virulence factors, which can be used to correlate with a patient’s clinical outcome to provide a more nuanced understanding of the complex interplay of virulence and host factors.

In this study, we aimed to include all cases of STEC in the southern region of the province of Alberta, Canada, from 2020 to 2022 to describe patient demographics and clinical outcomes and to correlate these outcomes with virulence factors identified by WGS.

## 2. Materials and Methods

Stool samples submitted to the South Zone in Alberta were cultured and tested for the presence of STEC using their local laboratory standardized method. All STEC strains isolated were submitted to ProvLab for serotyping and surveillance. In this study, a total of 107 isolates from 1 January 2020 to 17 October 2022 were included. Upon receipt of isolates, they were grown on blood agar plates and subject to WGS using the PulseNet standardized protocol (https://pulsenetinternational.org/protocols/wgs/, accessed on 17 September 2024). Briefly, the DNA was extracted using the DNA extraction MagaZorb^®^ DNA Mini-Prep Kit (Promega, Madison, WI, USA) using the KingFisher mL extractor (ThermoScientific, Ottawa, ON, Canada) according to the manufacturer’s instructions. Purified DNA was quantified using Qubit™ 1X dsDNA BR Assay Kit (ThermoFisher, Ottawa, ON, Canada), and Nextera XT DNA Library Prepkit (Illumina, San Diego, CA, USA) was used to prepare the library. Post PCR, AMPure XP bead-based reagent was used to clean up the amplified product, followed by DNA quantification with the Qubit™ 1X dsDNA High Sensitivity (HS) Assay Kit (Thermo Fisher, Ottawa, ON, Canada), and the sizing was checked using the Agilent Tape station (Agilent, Santa Clara, CA, USA). The DNA concentration was standardized before pooling, followed by loading onto the MiSeq instrument using the MiSeq reagent kit v3 (Ilumina, San Diego, CA, USA).

Serotyping was performed in silico from WGS data using ECTYPER (v1.0.0; https://github.com/phac-nml/ecoli_serotyping, accessed on 1 September 2023) [4]. Demographic and clinical outcome data were obtained through a review of the electronic health records. WGS data were analyzed using pathogenseq (v1.12; https://github.com/xiaoli-dong/pathogenseq, accessed on 1 March 2024). Briefly, the pathogenseq pipeline assessed sequence quality using FastQC (v.0.12.1; https://github.com/s-andrews/FastQC, accessed on 1 March 2023), fastp (v0.23.4) [5], and SeqKit (v2.6.0) [6]. De novo genome assemblies were created using shovill (v1.1.0; https://github.com/tseemann/shovill, accessed on 17 September 2024) with a minimum cutoff length of 300 base pairs (--minlen 300). Genome assembly quality was assessed using CheckM2 (v1.0.1; https://github.com/chklovski/CheckM2, accessed on 1 April 2023) and SeqKit (v2.6.0). Virulence gene analysis was performed using abriTAMR (v1.0.0; https://github.com/MDU-PHL/abritamr, accessed on 1 April 2023) and AMRFinderPlus with database version 2024-05-02.2 (v3.12.8) [7].

Shiga toxin *stx* subtyping was performed based on a comparison of the study *stx* sequences with that of the *stx* sequences obtained from the ABRicate workflow. The *stx* subtypes of STEC isolates were initially determined by ABRicate version 0.8.10 (https://github.com/tseemann/abricate, accessed on 16 November 2022) with the default parameters. Briefly, a *stx* subtyping database was created with ABRicate by including representative nucleotide sequences of all identified *stx1* and *stx2* subtypes from the Virulencefinder database. The assemblies were then searched against the *stx* subtyping database [8]. Further confirmation was performed using real-time PCR with primers and probes, as described by Zhi et al. (2019) [9].

Cluster analysis was performed using wgMLST within the BioNumerics v7.6.3 (BioMerieux, Saint-Laurent, QC, Canada) platform. A dendrogram was constructed with BioNumerics v7.6.3 using a categorical (values) similarity coefficient and an unweighted pair group method with an arithmetic mean (UPGMA) clustering algorithm.

## 3. Results

A total of 106 STEC isolates were recovered from 104 patients during the study period. The mean age was 22 years, with a range of 0 to 71 years, and 55 (52.9%) patients were female. A total of 19 of 104 were hospitalized (18.1%). Of these 19, 13 (68.4%) were hospitalized but did not develop HUS. Six patients (5.8% of the total) developed HUS, and three (50% of HUS cases) required dialysis. One hospitalized patient died within 30 days of initial stool sample submission; the cause of death could not be definitively assigned by chart review. Hospitalized patients without HUS had a median age of 25.5 years and a median duration of hospitalization of 3.5 days. HUS patients were predominantly female (5/6, 83.3%) with a median age of 2.5 years. Three cases were aged 1 year, and the others were 4, 17, and 60 years of age.

The most common serotype in all cases was O157:H7 (37, 34.9%), followed by O26:H11 (31, 29.2%), O121:H19 (9, 8.5%), O111:H8 (8, 7.5%) and O103:H2 (5, 4.7%). Of the HUS cases, 4/6 (66.7%) were O157:H7 alone, and 2/6 (33.3%) were mixed infections with O157:H7 and another non-O157 serotype (Table 1).

Virulence factor analysis demonstrated that certain genes, including *eae*, *espA*, *espB nleA*, and *tir*, were seen in ≥98% of all clinical STEC isolates. Several other genes were essentially absent (<2%) from all the STEC cases (*mchB*, *mchF*, *sta1*, and *tccP*). Several genes were absent in all O157 cases but present in varying frequencies in the non-O157 cases, including *cif*, *efa1*, *espF*, *espI*, *iucA*, *iucB*, *iucC*, *iucD*, *iutA*, *lpfA-O113*, *ybtP*, and *ybtQ.* Three genes were seen more commonly in O157 cases than in non-O157 cases (*etpD*, *iha*, *stxB2*).

Among the O157 cases, three genes (*espP*, *espX1*, *katP*) were seen more frequently in the HUS (100%, 100%, 100%) than in the hospitalized (71.4%, 85.7%, 71.4%) or non-hospitalized (87.5%, 83.3%, 87.5%) cases. Unfortunately, the case numbers were too low to determine statistical significance. Among the non-O157 cases, *lpfA* was less common in hospitalized cases (50%) than in non-hospitalized cases (83.6%). This virulence factor was not present in any of the O157 cases (Table 2).

The stx subtype patterns were investigated, and the *stx1a* was the most common stx subtype observed overall. Of the O157:H7 isolates from HUS patients, 100% of them carried one or two subtypes of *stx2*, including two with *stx1a*, *stx2a*, three with *stx2a*, *stx2c*, and one with the *stx2a* pattern. The combination of *stx2a/c* was more common in the patients with HUS (37.5%) compared to the hospitalized (14.3%) and non-hospitalized (3.5%) patients (Table 3).

Of the 37 O157:H7 isolates, there were 21 *stx1a* and *stx2a*, two *stx1a* and *stx2c*, six *stx2a*, and eight *stx2a* and *stx2c*. Of the 6 HUS cases, all 6 carried O157:H7 isolates with *stx2*. Two had *stx1a* and *stx2a*, one had *stx2a,* and three had *stx2a* and *stx2c*; 2 out of 6 additionally carried non-O157 serotypes, which were both *stx1a* alone.

Of 31 O26:H11 cases (Table 1), 28 (90.3%) were *stx1a,* and 3 (9.7%) were *stx1a*, *stx2a*. There were two hospitalized cases, and the HUS had a co-infection of O26:H11 (*stx1a*) and O157:H7 (*stx2a*). Another patient who developed HUS also had a co-infection of O103:H11 (*stx1a*) and O157:H7 (*stx2a* and *stx2c*).

Cluster analysis did not identify any outbreaks during the study period. For O157 serotypes, three clusters were identified, each including two cases. Two of these clusters were sets of pediatric siblings in which both individuals were hospitalized. O103:H11 had one cluster of two cases in which one patient aged one year developed HUS (this patient had a co-infection with O157:H7). The *stx* type in these O103:H11 cases was *stx1a*.

## 4. Discussion

Between 2020 and 2022 in southern Alberta, Canada, 107 STEC isolates were identified from 105 patients. A review of the corresponding clinical information found that 20 (19.0%) experienced severe clinical outcomes, including hospitalization alone (14, 13.3%), HUS (6, 5.7%), and death (1, 0.9%). The deceased patient died within 30 days of stool sample submission, although due to limited available information, the death could not be definitively attributed to STEC infection. HUS cases, as shown previously in the literature, were primarily found in young children less than 5 years of age [10]. HUS cases also had a high proportion requiring initiation of dialysis (3/6, 50%).

O157:H7 made up 34.6% of all the STEC isolated but accounted for 50% of all the hospitalized cases. A total of 100% (6/6) of HUS cases had an O157:H7 isolated; two of these cases had co-infections with non-O157 serotypes. Previous literature has likewise demonstrated significantly more hospitalizations and HUS cases with O157:H7 than non-O157 serotypes [11].

Whole genome sequencing data were able to identify the presence of specific virulence factors in these patients. Certain genes were found in >98% of all STEC clinical cases, regardless of clinical outcome, suggesting their importance to pathogenesis. These included *eae*, *espA*, *espB*, *nleA*, and *tir*. Several of these genes are carried on the LEE, which has been previously associated with clinically significant STEC types that cause attaching and effacing (AE) lesions of the colonic mucosa [12]. Several genes were found at very low frequencies in all of the STEC isolates, including *mchB*, *mchF*, *sta1*, *and tccP*. *sta1* is more commonly found in enterotoxigenic *E. coli* (ETEC) isolates [13]. Interestingly, *tccP* was found at very low levels in our study, though previous studies have shown this effector protein of the Type III secretion system to be found at very high frequency in clinical STEC isolates [14].

The plasmid pO157 is a known virulence factor of O157:H7 STEC that has been shown to be much more common in clinical isolates than those from bovine sources [15]. Some of the genes encoded here include *ehxA*, *etpC-O*, *espP*, *katP*, *toxB*, *ecf*, and *stcE*. Together, the pO157 contributes to bacterial colonization in cattle as well as adherence and survival in humans [16]. Consistent with this, we found *etp*D to be more common in O157 cases than non-O157. Additionally, we noted *espP* and *katP* to be found at higher frequency in O157 HUS cases than those that were hospitalized alone.

Overall, the most common Shiga toxin subtype profile was *stx1a* alone. The HUS cases all carried an isolate with *stx2*. In particular, the combination of subtypes a and c, represented by *stx2a* and *stx2c,* appeared to be over-represented in HUS cases in comparison to hospitalized and non-hospitalized cases (Table 3). This is in keeping with previous literature [2,17].

Strengths of this work include 2 years of clinical metadata of STEC isolates with complete accompanying WGS data. Limitations include the relatively small number of HUS cases identified (6/105). Hospitalization is a subjective measure of the severity of disease, as practices and confounding factors may vary.

Overall, the number of STEC isolates included in this study was insufficient to demonstrate any significant differences in virulence factor profiles for different clinical outcomes apart from what may be explained by differences in serotype and *stx* profile.

This work adds to the body of literature describing the association between specific STEC virulence genes and clinical outcomes. Identification of Shiga toxins and their subtypes alone is not sufficient to predict the severity of disease in patient cases. Clinical disease is related to a complex interplay of host, organism, and environmental factors. The association of the key organism factors on disease severity surveyed in this study includes serotype, *stx* type, and virulence gene profiles as well. Further research may someday allow for more nuanced prognostication in individual patient cases based on the pattern of virulence factors present in an individual’s strain of STEC.

## Figures and Tables

**Table 1:** Number (percentage) of different STEC serotypes by clinical outcome.

Serotype	Non-Hospitalized/ Non-HUS n (%)	Hospitalized n (%)	HUS n (%)	Total n (%)
O157:H7	24 (28.2)	7 (53.8)	6 (75.0)	37 (34.9)
O26:H11	28 (32.9)	2 (15.4)	1 ^b^ (12.5)	31 (29.2)
O121:H19	7 (8.2)	2 (15.4)	0	9 (8.5)
O111:H8	8 (9.4)	0	0	8 (7.5)
O103:H2	5 (5.9)	0	0	5 (4.7)
O103:H11	2 (2.4)	0	1 ^b^ (12.5)	3 (2.8)
O123/O186:H2	0	1 (7.7)	0	1 (0.9)
O177:H25	1 (1.2)	1 (7.7)	0	2 (1.9)
Other ^c^	10 (11.8)	0	0	10 (9.4)
Total	85 (100)	13 (100)	8 ^a^ (100)	106 (100)

^a^ A total of 8 STEC isolates were retrieved from 6 cases of HUS. A total of 4/6 cases were O157:H7 alone, 1 was O157:H7 with O103:H11, and 1 was O157:H7 with O26:H11; these 2 non-O157 co-infections are indicated by ^b^ in the table. ^c^ Other serotypes included: O71:H11, O168:H8, O5:H9, O156:H25, O98:H21, O45:H2, O145:H-, (2) O177:H25, O rough:H2.

**Table 2:** Number (percentage) of cases possessing each virulence factor by clinical outcome.

	O157 (n = 37)			non-O157 (n = 67 *)	
Gene	Non-Hospitalized, Non-HUS (n = 24)	Hospitalized, Non-HUS (n = 7)	HUS (n = 6)	Non-Hospitalized, Non-HUS (n = 61)	Hospitalized, Non-HUS (n = 6)
*astA*	5 (20.8)	2 (28.6)	2 (33.3)	8 (13.1)	2 (33.3)
*cif*	0 (0)	0 (0)	0 (0)	47 (77)	4 (66.7)
*eae*	24 (100)	7 (100)	6 (100)	60 (98.4)	6 (100)
*efa1*	0 (0)	0 (0)	0 (0)	54 (88.5)	5 (83.3)
*ehxA*	21 (87.5)	5 (71.4)	4 (66.7)	55 (90.2)	6 (100)
*espA*	24 (100)	7 (100)	6 (100)	60 (98.4)	6 (100)
*espB*	24 (100)	7 (100)	6 (100)	60 (98.4)	6 (100)
*espF*	0 (0)	0 (0)	0 (0)	48 (78.7)	5 (83.3)
*espI*	0 (0)	0 (0)	0 (0)	14 (23)	2 (33.3)
*espJ*	21 (87.5)	7 (100)	6 (100)	55 (90.2)	5 (83.3)
*espK*	23 (95.8)	7 (100)	6 (100)	57 (93.4)	5 (83.3)
*espP*	21 (87.5)	5 (71.4)	6 (100)	42 (68.9)	5 (83.3)
*espX1*	20 (83.3)	6 (85.7)	6 (100)	41 (67.2)	3 (50)
*etpD*	22 (91.7)	6 (85.7)	5 (83.3)	3 (4.9)	1 (16.7)
*fdeC*	24 (100)	6 (85.7)	6 (100)	52 (85.2)	6 (100)
*iha*	24 (100)	7 (100)	6 (100)	43 (70.5)	5 (83.3)
*iss*	2 (8.3)	1 (14.3)	0 (0)	36 (59)	4 (66.7)
*iucA*	0 (0)	0 (0)	0 (0)	42 (68.9)	5 (83.3)
*iucB*	0 (0)	0 (0)	0 (0)	45 (73.8)	5 (83.3)
*iucC*	0 (0)	0 (0)	0 (0)	45 (73.8)	5 (83.3)
*iucD*	0 (0)	0 (0)	0 (0)	45 (73.8)	5 (83.3)
*iutA*	0 (0)	0 (0)	0 (0)	45 (73.8)	5 (83.3)
*katP*	21 (87.5)	5 (71.4)	6 (100)	33 (54.1)	4 (66.7)
*lpfA*	0 (0)	0 (0)	0 (0)	51 (83.6)	3 (50)
*lpfA1*	24 (100)	7 (100)	6 (100)	1 (1.6)	0 (0)
*lpfA2*	24 (100)	7 (100)	6 (100)	0 (0)	0 (0)
*lpfA-O113*	0 (0)	0 (0)	0 (0)	53 (86.9)	5 (83.3)
*mchB*	0 (0)	0 (0)	0 (0)	1 (1.6)	0 (0)
*mchF*	0 (0)	0 (0)	0 (0)	1 (1.6)	0 (0)
*nleA*	24 (100)	7 (100)	6 (100)	60 (98.4)	6 (100)
*nleB*	23 (95.8)	7 (100)	6 (100)	57 (93.4)	6 (100)
*nleB2*	23 (95.8)	6 (85.7)	5 (83.3)	3 (4.9)	1 (16.7)
*nleC*	24 (100)	7 (100)	6 (100)	51 (83.6)	5 (83.3)
*sta1*	0 (0)	0 (0)	0 (0)	1 (1.6)	0 (0)
*stxA1*	18 (75)	3 (42.9)	2 (33.3)	51 (83.6)	4 (66.7)
*stxA2*	21 (87.5)	5 (71.4)	3 (50)	17 (27.9)	2 (33.3)
*stxB1*	18 (75)	3 (42.9)	2 (33.3)	51 (83.6)	4 (66.7)
*stxB2*	24 (100)	7 (100)	6 (100)	17 (27.9)	2 (33.3)
*tccP*	0 (0)	0 (0)	0 (0)	2 (3.3)	0 (0)
*tir*	24 (100)	7 (100)	6 (100)	60 (98.4)	6 (100)
*toxB*	7 (29.2)	3 (42.9)	0 (0)	28 (45.9)	3 (50)
*ybtP*	0 (0)	0 (0)	0 (0)	33 (54.1)	2 (33.3)
*ybtQ*	0 (0)	0 (0)	0 (0)	33 (54.1)	2 (33.3)

*astA*, heat-stable enterotoxin EAST1; *cif*, type III secretion system effector Cif; *eae*, intimin type theta; *efa1*, lymphostatin Efa1/LifA; *ehxA*, enterohemolysin EhxA; *espA*, type III secretion system LEE translocon filament protein EspA; *espB*, type III secretion system LEE translocon pore-forming subunit EspB; *espF*, type III secretion system LEE effector EspF; *espI*, serine protease autotransporter EspI; *espJ*, type III secretion system effector ADP-ribosyltransferase EspJ; *espK*, type III secretion system effector EspK; *espP*, serine protease autotransporter EspP; *espX1*, type III secretion system effector EspX1; *etpD*, variant type II secretion system secretin EtpD; *fdeC*, inverse autotransporter adhesin FdeC; *iha*, bifunctional siderophore receptor/adhesin Iha; *iss*, increased serum survival lipoprotein Iss; *iucA*, aerobactin synthase IucA; *iucD*, NADPH-dependent L-lysine N(6)-monooxygenase IucD; *iutA*, ferric aerobactin receptor IutA; *katP*, catalase/peroxidase KatP; *lpfA*, long polar fimbria major subunit LpfA; *lpfA1*, long polar fimbria major subunit LpfA1; *lpfA2*, long polar fimbria major subunit LpfA2; *lpf-O113*, long polar fimbria major subunit LpfA-O113; *mchB*, microcin H47; *mchF*, microcin H47 export transporter peptidase/ATP-binding subunit MchF; *nleA*, type III secretion system effector NleA; *nleB*, type III secretion system effector arginine glycosyltransferase NleB; *nleB2*, type III secretion system effector arginine glycosyltransferase NleB2; *nleC*, type III secretion system effector zinc metalloprotease NleC; *sta1*, heat-stable enterotoxin ST-I group a; *tccP*, Tir-cytoskeleton coupling protein TccP2; *tir*, type III secretion system LEE translocated intimin receptor Tir; *toxB*, toxin B; *ybtP*, yersiniabactin ABC transporter ATP-binding/permease protein YbtP; *ybtQ*, yersiniabactin ABC transporter ATP-binding/permease protein YbtQ. * 2/6 HUS cases were co-infections with O157:H7 and another non-O157 serotype (O103:H11 and O26:H11). The two non-O157 HUS cases have been excluded from this analysis.

**Table 3:** Number (percentage) of cases with each *stx* subtype pattern by clinical outcome.

*stx* Subtype Pattern	NON-Hospitalized, Non-HUS n (%)	Hospitalized n (%)	HUS * n (%)	Total n (%)
*stx1a*	44 (51.8)	4 (35.7)	0 (0)	48 (45.7)
*stx1a* and *stx2a*	23 (27.1)	3 (21.4)	2 (33.3)	28 (26.7)
*stx1a* and *stx2c*	2 (2.4)	0 (0)	0 (0)	2 (1.9)
*stx2a*	11 (12.9)	3 (21.4)	1 (16.7)	15 (14.3)
*stx2a* and *stx2c*	3 (3.5)	2 (14.3)	3 (50.0)	8 (7.6)
*stx2c*	1 (1.2)	1 (7.1)	0 (0)	2 (1.9)
*stx2d*	1 (1.2)	0 (0)	0 (0)	1 (0.9)

* 2/6 HUS cases were co-infections with O157:H7 and another non-O157 serotype (O103:H11 and O26:H11). For these 2 cases, the *stx* type of the O157 serotype is presented. The non-O157 isolates from both of the co-infections were *stx1a* alone.

## Data Availability

The original data presented in the study are openly available in NCBI at https://www.ncbi.nlm.nih.gov/. Reference numbers: SAMN41568052-SAMN41568157.
